# Anatomical Determinants of Rare Congenital Cardiac Disorders: A Narrative Review

**DOI:** 10.7759/cureus.105966

**Published:** 2026-03-27

**Authors:** Kumar Sambhav, Naveed Mohsin, Rajeshwar Yadav, Ajmal Ali, Santosh Mohanlal Modani, Sunil Pathak

**Affiliations:** 1 Department of Anatomy, All India Institute of Medical Sciences, Bilaspur, Bilaspur, IND; 2 Department of Internal Medicine, Sher-i-Kashmir Institute of Medical Sciences Soura, Srinagar, IND; 3 Department of Cardiothoracic and Vascular Surgery (CTVS), Banaras Hindu University, Varanasi, IND; 4 Department of Medicine, Government Medical College Kottayam, Kottayam, IND; 5 Department of Interventional Cardiology, Dr Santosh Cardiac Care Centre, Warangal, IND; 6 Department of Pediatrics, Dr. N.D. Desai Faculty of Medical Science and Research, Dharmsinh Desai University, Nadiad, IND

**Keywords:** aortic arch anomalies, cardiac morphology, congenital heart disease, conotruncal malformations, ventricular physiology

## Abstract

Rare congenital cardiac disorders emerge from early developmental disturbances that reshape chamber geometry, alter arterial and venous alignment and modify valvular architecture, creating complex structural patterns with distinct physiological consequences. A central rationale for this narrative review is the need to unify scattered morphological insights to support a clearer interpretation of these uncommon conditions across varied clinical environments. This narrative review focuses on integrating the principal anatomical determinants that shape the presentation of rare congenital cardiac anomalies, linking developmental events with structural configurations that influence circulatory behavior, physiological adaptation and long-term functional stability. Literature published from 2015 to 2025 was examined through targeted searches across major scientific databases, selecting studies that offered detailed structural, embryological or imaging-based perspectives relevant to these disorders. The content integrates current understanding of looping deviations, conotruncal malformations, anomalous venous pathways, valvular morphogenesis defects, laterality disturbances, vascular rings and advanced imaging contributions, outlining how each category introduces distinctive shifts in hemodynamic patterns. This narrative review underscores the importance of precise morphological evaluation for early recognition, informed procedural planning, optimized intervention strategies, enhanced risk stratification and comprehensive longitudinal assessment. Consolidated anatomical insight strengthens diagnostic confidence and improves clinical decision-making for individuals presenting with rare congenital cardiac abnormalities. Such integrated knowledge ultimately supports the development of more refined and individualized management pathways.

## Introduction and background

Congenital cardiac anomalies represent a heterogeneous group of structural abnormalities arising from disruptions in cardiac morphogenesis (the process by which the embryonic heart forms and organizes its chambers and vessels), with a subset of complex or uncommon variants considered rare in clinical practice [1]. The developing heart goes through a cycle of mechanisms that involve looping of the primitive tube, formation of the atrial and ventricular chambers, remodeling of the atrioventricular canal, formation of outflow tracts and maturation of the valvular apparatus, which are key developmental stages that establish the normal structural framework required for effective postnatal circulation [2]. Disturbances in these coordinated processes may alter the spatial relationships among chambers, valves, coronary arteries and the great vessels. These changes generate complicated structural patterns, which alter the circulation of neonates at the very beginning of extra-uterine life and may clinically manifest as cyanosis, heart failure, or circulatory instability depending on the underlying defect [3]. Examples of the disorders that result in atrioventricular septal defects, truncus arteriosus and other disorders of the great vessels and abnormal laterality of the great vessels (disturbances in the normal left-right arrangement of cardiovascular structures) demonstrate the broad morphological range of conditions that occur as a result of cardiac development disorders [4]. All of them have unique architectural elements that determine the patterns of oxygenation, the condition of ventricular loading, the pressure gradients, and the possibility of correction by a surgical intervention or catheter-based therapy [5]. The clinical phenotype in different individuals frequently varies significantly, and it is indicative of the sensitivity of the developing heart to small changes in spatial positioning or tissue reorganization [6]. Circulatory adaptation in early life is extremely reliant on the equilibrium between the structural integrity and the postnatal physiological demands, and thus, a proper delineation of anatomy is crucial to timely clinical stabilization and appropriate early clinical management [7].

Modern imaging modalities have increased the knowledge of the structural landscape of these disorders and play a central role in clinical diagnosis and treatment planning [8]. Currently, high-resolution echocardiography, fetal cardiac imaging, multidetector computed tomography and cardiac magnetic resonance provide detailed visualization of chamber geometry, arterial alignment, patterns in coronary origin and valvular anatomy [5]. Such technologies unveil the structural peculiarities that were not noticed before and allow identifying the variant forms that cannot be included in classical characterizations [3]. Innovations in reconstruction methods also increase spatial knowledge of complex relationships between anatomy, allowing clinicians to better appreciate the three-dimensional organization of cardiac structures during diagnostic evaluation and pre-procedural planning [7]. This kind of imaging goes beyond mere identification of abnormalities and helps in a more detailed depiction of functional effects by depicting flow courses, chamber interrelationships and vascular positions that influence circulatory performance [9]. Even though the level of advances has been successful in improving the level of precision in diagnosis, interpretation still relies on a clear understanding of the anatomical determinants that will inform the functioning behavior of every condition [2]. Structural mapping remains essential in early detection of rare congenital anomalies, risk assessment and the plan of procedure [10].

There are still considerable gaps that restrain a full comprehension of these disorders. Most anatomical descriptions do not relate to the sequence of development that gives rise to each malformation, making it difficult to be clear about how this or that structural form came to be [11]. Phenotypic variability generates intermediate forms, which undermine traditional classification and bring inconsistency to the meaning of diagnosis between clinical environments [12]. The absence of established morphological terms further disrupts communication and makes it difficult to compare the structural results [5]. The combination of anatomy with genetic, molecular and laterality modulations has not been sufficiently studied, yet it has been demonstrated that these mechanisms play an important role in cardiac patterning and the emergence of congenital anomalies [9]. Moreover, the correlations between the structural determinants and the long-term clinical outcomes remain incompletely synthesized, which restricts the predictive power in the decision-making process and decreases the ability of advanced imaging to gain interpretations [7].

Improved characterization of morphological determinants enhances clinical recognition of atypical congenital presentations and supports structured evaluation of disease severity [10]. Neonatal physiology is extremely sensitive to the variation of architecture, and detection of an early instance is reliant on the interpretation of structural indicators that characterize circulatory behavior [2]. The use of surgical and catheter-based approaches is also based on accurate outlining of chamber sizes, relationships between outflow and valve morphology and coronary distribution during procedural planning [13]. A consolidated morphological perspective can therefore improve diagnostic accuracy by linking specific anatomical configurations with predictable physiological responses during circulatory transition in infancy and early childhood. The elucidation of factors of ventricular adaptation and any possible progressive obstruction and valve dysfunction, or vascular remodeling, is also detailed through longitudinal structural assessment. Enhancing the anatomical understanding will eventually improve the efficacy of the multidisciplinary care pathways and richness of the clinical paradigm, in which rare congenital cardiac maladies are perceived and treated.

Objectives of the review

This review aims to consolidate key anatomical determinants underlying rare congenital cardiac disorders, integrate developmental pathways with structural interpretation and highlight how detailed morphological analysis enhances diagnostic precision and guides therapeutic planning in contemporary cardiovascular practice.

## Review

Methodology

Literature Search Strategy

A selective literature review was conducted through PubMed, Scopus, Web of Science and Google Scholar to identify articles published between 2015 and 2025 addressing anatomical determinants of rare congenital cardiac disorders. Searches were performed using combinations of relevant keywords and Medical Subject Headings (MeSH) terms, including “congenital cardiac anomalies,” “cardiac morphogenesis,” “conotruncal malformations,” “vascular rings,” “coronary artery anomalies,” “cardiac structural defects,” and “cardiac imaging.” Boolean operators (AND/OR) were used to refine search combinations and improve retrieval of relevant records. Bibliographies of the most significant publications were also manually screened to identify additional relevant studies.

Study Selection and Screening Process

Peer-reviewed articles published in English between 2015 and 2025 with detailed descriptions of structural anatomy, embryological interpretation, anatomical assessment through imaging or clinically relevant morphological correlations were considered eligible. Titles and abstracts were initially screened for relevance, followed by full-text evaluation of potentially eligible studies to confirm inclusion. Duplicate records identified across databases were removed during the screening process. Case reports, observational studies and reviews were considered when they provided substantive anatomical or morphological insights relevant to rare congenital cardiac anomalies.

Eligibility Criteria

Inclusion criteria: Studies were included if they were peer-reviewed publications published between 2015 and 2025, provided detailed anatomical, embryological or imaging-based analysis of rare congenital cardiac anomalies and presented morphological interpretations with clinical relevance.

Exclusion criteria: Studies were excluded if they focused on animal models without clear translational relevance to human cardiac anatomy, addressed common congenital heart conditions without relevance to rare variants, lacked sufficient anatomical or structural description or investigated genetic or molecular mechanisms without structural or anatomical correlation. Articles published outside the 2015-2025 timeframe, unavailable in full text or with unclear methodological description were also excluded.

Evidence Consideration and Synthesis

Because this work follows a narrative review framework, formal risk-of-bias tools were not applied; however, studies were critically assessed for anatomical relevance, methodological clarity and contribution to morphological interpretation. The assessment of potential bias and study relevance was independently performed by two authors, and any discrepancies were resolved through discussion to reach consensus. The final selection emphasized studies contributing to a consolidated morphology-based synthesis linking developmental mechanisms with clinically relevant structural patterns.

Morphological impact of looping and septation defects

Cardiac Looping Abnormalities

Septation and cardiac looping are key events in cardiogenesis, which define the spatial orientation and chamber organization that are necessary to coordinate circulatory activity [14]. The primitive heart tube becomes bent and twisted to the right, leading to the orientation of the atrial and ventricular spaces and the beginning of the separation of the chambers [9]. The perturbations at this phase cause positional shifts between forming cardiac segments, creating misalignments that are the cause of several unusual congenital cardiac conditions [11]. Abnormal looping leads to discordant atrioventricular connections, great vessel malposition or ventricular outflow displacement, which impacts complicated circulatory patterns with severe implications on the postnatal hemodynamics [2].

Septation Defects and Structural Consequences

Separation in the form of interactions between endocardial cushions, conotruncal ridges and myocardial growth centers is orchestrated. Incomplete or aberrant development of these areas results in the formation of defects, including anomalies of the atrioventricular septation, misaligned ventricular septal defects, double outlet right ventricle and conotruncal dysregulation [15]. All conditions have a characteristic pattern of architecture that is dictated by the extent and orientation of the deviation of the septa [8]. Disturbance of atrial or ventricular partitioning changes intracardiac flow patterns, resulting in abnormal patterns of shunt, unbalanced ventricular loading and changed pressure relationships [13]. The abnormalities also affect the systemic and pulmonary circulation and usually display early cyanosis, impaired ventricular performance or complicated outflow tract blockage [14].

Conotruncal Septation and Rare Congenital Variants

The relevance of conotruncal septation abnormalities is of special significance in rare congenital forms, because conal rotation, crest fusion and truncal remodeling form the basis upon which aortic and pulmonary separation is developed [7]. These malformations of processes lead to structural configurations of truncus arteriosus, interrupted aortic arch or subpulmonic malalignment [2]. Those exceptions reflect how delicate early developmental morphogenetic processes were to small developmental changes [10].

Morphological Interpretation and Developmental Correlation

**Figure 1:**
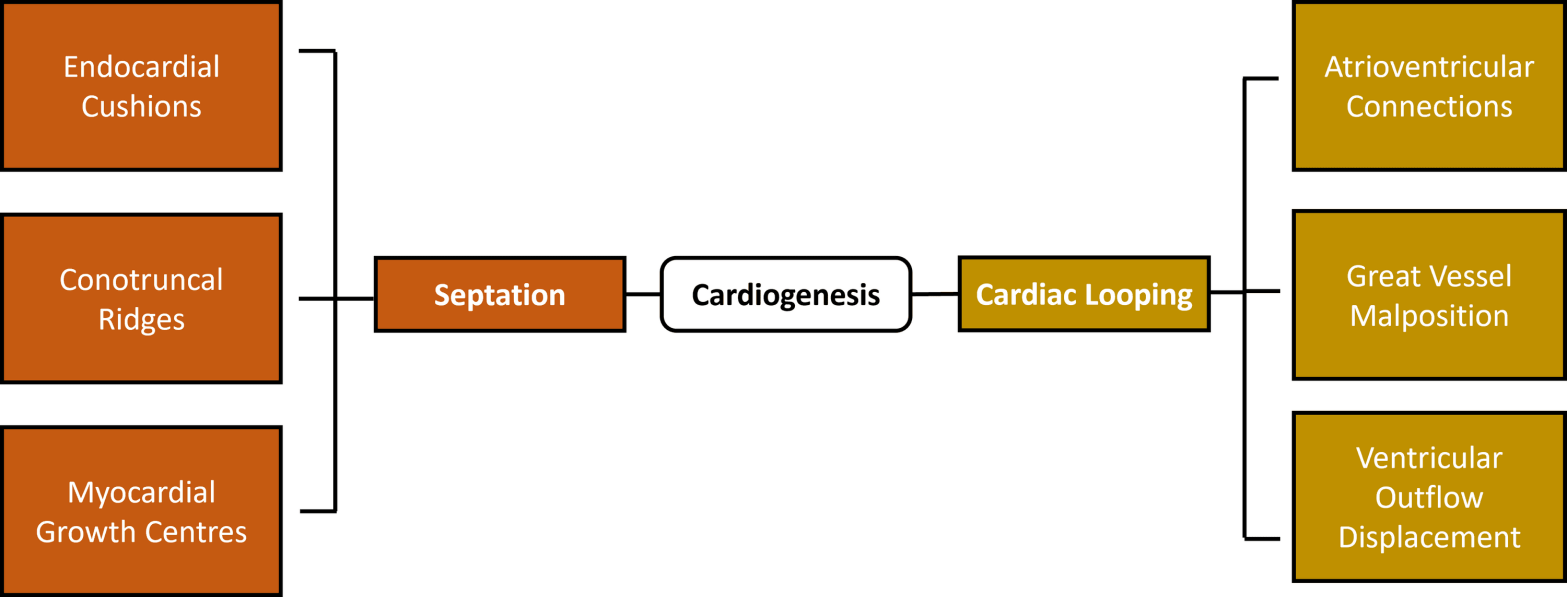
Cardiac looping and septation during embryogenesis, showing how developmental disruption leads to structural congenital cardiac anomalies Created by authors using Microsoft PowerPoint (Microsoft Corporation, Redmond, WA, USA).

Complete knowledge of looping and septation defects enhances proper morphological interpretation of the disturbance, which increases the confidence of the diagnosis and directs the clinical evaluation of unusual congenital heart defects [16]. Figure 1 shows the key embryological processes of cardiogenesis, highlighting septation and cardiac looping and their associated structural outcomes.

Malformations of the conotruncal region and outflow tracts

Development of the Conotruncal Region

The partitioning and alignment of the arterial trunks are controlled by the conotruncal development that directs the separation of the systemic and the pulmonary outflow [17]. The process involves coordinated rotation, septation and remodeling, which is regulated by interactions between the neural crest elements, conal ridges and truncal structures [14]. Disruptions at these stages bring about changes in the spatial relationship between the ventricles and great arteries and result in a range of unusual birth defects in the heart with profound circulatory effects. The structural arrangement of every anomaly indicates the extent of malformation, the direction of conal displacement, and the completeness of truncal splitting [11].

Structural Consequences of Conotruncal Malformations

Distorted conotruncal geometry alters ventricular loading, arterial flow distribution and the feasibility of corrective surgery, which highlights the clinical importance of accurate morphological interpretation [9]. Double outlet right ventricle (DORV) is characterized by both arterial trunks originating mainly from the right ventricle and is commonly accompanied by misaligned septation and uneven subarterial obstruction [15]. Truncus arteriosus results from failure to form a complete aortopulmonary septum, allowing one arterial trunk in which systemic, pulmonary and coronary circulations arise [13]. Interrupted aortic arch occurs as a result of regression or failed fusion of the fourth pharyngeal arch component and causes discontinuity of the aortic lumen with severely compromised systemic flow [3].

Morphological Patterns and Structural Determinants

Both conditions depict distinctive structural determinants developed through early deviations of development. These anomalies illustrate how alterations in conotruncal rotation, septation and arterial alignment can generate diverse structural configurations that influence postnatal circulatory dynamics. Table 1 shows the major structural features and signs of major conotruncal malformations.

**Table 1:** Structural features associated with major conotruncal malformations Compiled by the authors based on information synthesized from [4,13,16]. The table is not directly reproduced from any single external source; therefore, permission from original publishers was not required. DORV: double outlet right ventricle, RV: right ventricle, VSD: ventricular septal defect, TA: truncus arteriosus, IAA: interrupted aortic arch, PA: pulmonary artery.

Malformation	Primary Structural Change	Ventricular-Arterial Relation	Hemodynamic Impact	Imaging Cues	References
DORV	Predominant arterial origin from the RV	Variable alignment; VSD-dependent	Mixed or obstructed flow patterns	RV-dominant arterial emergence	[13]
TA	Absence of aortopulmonary septum	Single outflow trunk over VSD	Parallel systemic-pulmonary flow	Common trunk with branch PA origins	[16]
IAA	Discontinuity of the aortic lumen	Normal ventriculo-arterial connection	Severe systemic outflow reduction	Absence of arch continuity	[4]

Anomalous venous connections and systemic-pulmonary flow disruptions

Pulmonary Venous Development and Anomalies

Anomalous venous formation occurs due to abnormal development during the incorporation of pulmonary and systemic venous pathways into the atria [18]. Normally, the pulmonary venous plexus connects to the left atrium through gradual absorption of the common pulmonary venous channel [17]. Disruption of this developmental sequence redirects pulmonary venous return toward systemic venous structures, altering oxygenation and intracardiac flow patterns [3].

Total anomalous pulmonary venous connection occurs when the entire pulmonary venous system drains into the right atrium or its tributaries, resulting in complete mixing of oxygenated and deoxygenated blood [7]. Partial anomalous pulmonary venous connection develops when one or more pulmonary veins drain into systemic veins rather than the left atrium, leading to reduced effective pulmonary venous return to the left atrium [19]. The degree of shunting and the anatomical position of the anomalous channels determine the severity of cyanosis, right-sided volume loading and the risk of pulmonary hypertension [20].

Systemic Venous Developmental Variants

Additional complexity arises from anomalies of the systemic venous system. Abnormal development of the cardinal, subcardinal or supracardinal veins may produce persistence of the left superior vena cava, interrupted inferior vena cava with azygos continuation or atypical hepatic venous drainage patterns [15]. These variations modify systemic venous return, influencing preload conditions, atrial geometry and the cardiac response to circulatory stress [6].

Hemodynamic Consequences and Clinical Relevance

Combined pulmonary and systemic venous anomalies create distinctive flow dynamics at the cardiopulmonary interface, producing abnormal admixture patterns, chamber dilation and redistribution of pressure across the cardiopulmonary circulation [19]. Accurate identification of venous architecture is essential, as these variants influence procedural access routes, the feasibility of cavopulmonary connections and postoperative hemodynamic stability [8]. Clear delineation of venous drainage pathways, obstruction sites and atrial connections improves clinical interpretation and guides intervention strategies in complex congenital heart disease [20].

Coronary artery variants and their role in rare congenital syndromes

Development of the Coronary Arterial System

Growth of the coronary arteries depends on the coordinated development of the epicardial vascular network, vascular buds and the aortic sinuses, forming a system that supplies oxygenated blood to the myocardium with precise spatial organization [21]. Disruptions during this developmental process can produce structural coronary variants that influence myocardial perfusion, ventricular function and susceptibility to ischemia [19].

Structural Variants of Coronary Arteries

Alterations in the course and caliber of coronary arteries may arise from anomalous coronary origin, abnormal sinuosity, interarterial courses between major vessels or the presence of a single coronary trunk [17]. These configurations can predispose the coronary arteries to external compression, turbulent flow or dynamic narrowing during periods of increased circulatory demand [22].

Coronary Variants in Rare Congenital Conditions

Several rare congenital syndromes include coronary variants within their structural pattern. Anomalous origin of the left coronary artery from the pulmonary artery (ALCAPA) diverts poorly oxygenated blood toward the left ventricular myocardium, leading to early ventricular dysfunction and potential circulatory collapse [12]. Single coronary artery associated with conotruncal malformations may supply the entire myocardium from a single arterial source, influencing global myocardial perfusion patterns [7]. Coronary artery fistulas create abnormal communications between coronary vessels and cardiac chambers or great arteries, altering regional blood flow distribution and reducing effective myocardial perfusion [20]. The clinical impact of these variants depends on the origin, pathway and termination of the affected coronary artery [23].

Clinical Significance and Structural Assessment

Accurate identification of coronary artery variants is essential for morphological interpretation and clinical management. Computed tomography angiography and cardiac magnetic resonance imaging provide detailed visualization of the coronary origin, course and their relationship to the aortic root and surrounding structures [10]. Recognition of these variants supports appropriate clinical decision-making, helps maintain hemodynamic stability and guides the selection of interventional strategies. Table 2 shows significant coronary variants with structural properties and functional implications.

**Table 2:** Key coronary variants and associated clinical features Compiled by the authors based on information synthesized from [5,18,23]. The table is not directly reproduced from any single external source; therefore, permission from original publishers was not required. ALCAPA: anomalous left coronary artery from the pulmonary artery, PA: pulmonary artery, LV: left ventricle, SCA: single coronary artery, CAF: coronary artery fistula.

Variant	Primary Structural Deviation	Coronary Course Pattern	Functional Impact	Imaging Indicators	References
ALCAPA	Origin from PA	Retrograde or collateral-dependent	LV ischemia and dysfunction	Dilated collaterals; PA-origin vessel	[23]
SCA	Single origin from the aorta sinus	Single trunk supplying the entire myocardium	Global perfusion vulnerability	Solitary root-origin artery	[18]
CAF	Coronary-chamber or vessel fistulous link	Variable tortuous pathway	Steal-induced perfusion reduction	Dilated feeding vessel with runoff	[5]

Structural determinants of single ventricle physiology

Developmental Basis of Single Ventricle Physiology

Single ventricle physiology is a congenital condition in which only one ventricular chamber is functionally capable of supporting either systemic or pulmonary circulation, placing substantial demands on myocardial performance and circulatory adaptation [24]. The condition arises from developmental abnormalities that restrict ventricular primordium growth or distort inflow and outflow pathways [5].

Morphological Patterns of Ventricular Hypoplasia

Structural patterns such as hypoplastic left heart syndrome show marked underdevelopment of the left ventricular cavity, mitral apparatus and aortic outflow, forcing systemic circulation to depend on a dominant right ventricle [20]. Conversely, hypoplastic right heart configurations develop from inadequate formation of the tricuspid valve or right ventricular outflow tract, resulting in reliance on the dominant left ventricle for forward circulation. These conditions display distinct chamber asymmetry, differences in myocardial mass distribution and variations in atrioventricular valve competence [18].

Hemodynamic Adaptation in Single Ventricle Circulation

Circulatory dynamics in single ventricle physiology depend on the balance between systemic and pulmonary vascular resistance because both circulatory pathways are supplied by a single functional chamber [7]. Excessive pulmonary blood flow leads to progressive ventricular dilation, imbalance in systemic oxygen delivery and increased metabolic demand. In contrast, reduced pulmonary blood flow results in hypoxemia, diminished ventricular preload and impaired systemic organ perfusion [19]. The interaction between vascular loading conditions and intrinsic ventricular structure determines overall circulatory stability [15].

Morphological Determinants of Long-Term Physiology

Key structural determinants, including ventricular geometry, atrioventricular valve competence, outflow tract patency and associated septation defects, shape the long-term evolution of single ventricle physiology [5]. These anatomical features influence the efficiency of circulation and the progression of ventricular functional adaptation over time.

Implications for Surgical Palliation

Distinct structural pathways guide staged surgical palliation, including systemic-pulmonary shunting, cavopulmonary connection and eventual total cavopulmonary circulation [11]. Accurate characterization of these anatomical determinants supports optimal timing of interventions and improves interpretation of ventricular performance changes during the progression of single ventricle physiology [25].

Valvular morphogenesis defects in rare congenital conditions

Development of Cardiac Valves

Valvular morphogenesis follows a regulated sequence that forms the atrioventricular and semilunar valves through endocardial cushion transformation, extracellular matrix remodeling and progressive leaflet stratification [26]. Disruptions during these developmental stages alter cusp geometry, supporting structures or annular configuration, producing distinct patterns of inflow or outflow dysfunction [21].

Atrioventricular Valve Malformations

Mitral valve malformations often arise from incomplete leaflet separation, abnormal chordal arrangement or restricted commissural formation [9]. These structural changes limit leaflet mobility and may produce inflow obstruction or regurgitant flow, increasing left atrial pressure and altering ventricular loading conditions [18]. Tricuspid valve anomalies may develop from inadequate delamination of the mural leaflet, malposition of papillary muscles or excessive apical displacement. These abnormalities contribute to regurgitation, right atrial enlargement and altered right ventricular mechanics [21].

Semilunar Valve Abnormalities

Pulmonary valve defects represent a different group of developmental deviations involving cusp dysplasia, asymmetric cusp fusion, or poorly developed interleaflet triangles [7]. Such alterations can lead to valvular stenosis or incomplete leaflet coaptation, affecting right ventricular ejection and pulmonary arterial pressure distribution [13]. Interactions between valve structure and adjacent conal or subvalvular regions may further modify hemodynamics, producing combined patterns of obstruction, regurgitation or mixed lesions [4].

Morphological Determinants and Clinical Relevance

**Figure 2:**
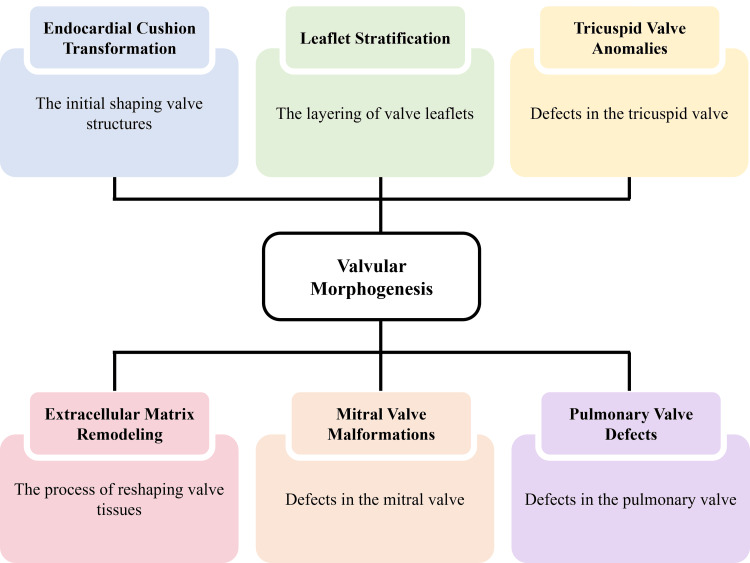
Stages of valvular morphogenesis and representative structural defects resulting from disrupted valve development Created by authors using Microsoft PowerPoint (Microsoft Corporation, Redmond, WA, USA).

The severity of these conditions depends on the degree of leaflet thickening, annular hypoplasia and structural continuity between the valve and surrounding myocardium [16]. Clear identification of these structural characteristics is important for accurate morphological interpretation and therapeutic planning [27]. Figure 2 shows the major developmental stages of valvular morphogenesis and the associated structural defects arising from disruptions in each process.

Atrioventricular junction malformations

Development of the Atrioventricular Junction

The development of the atrioventricular canal requires precise fusion, remodeling and resorption of the endocardial cushions at the atrioventricular junction [24]. These cushions form the septal components of the atrioventricular valves and help separate the right and left inflow tracts [28]. Disturbances during this developmental stage disrupt the alignment of the atrial septum, ventricular septum and atrioventricular annuli, producing a spectrum of atrioventricular canal defects with characteristic structural features [10]. Imbalance in the growth of the superior and inferior cushions can lead to persistence of a common atrioventricular junction, allowing communication between chambers and formation of a single annular valve structure [18]. This configuration alters inflow geometry, redistributes valvular tissue and may generate regurgitant or restrictive flow due to malformed leaflets [13].

Structural Variants of Atrioventricular Canal Defects

Partial fusion of the lateral cushion extensions contributes to the formation of valve leaflets but may also produce clefts in the valvular tissue and shared subvalvular support structures [10]. These anatomical features influence the direction of regurgitant jets, ventricular loading conditions and the degree of imbalance between the atrioventricular valves [22]. Junctional abnormalities may also result from misalignment between the inlet septum and muscular ventricular septum. Such defects produce inlet ventricular septal defects that can extend toward the outflow tract or apical regions, leading to left-to-right shunting and progressive ventricular dilation [29].

Conduction System Involvement

Structural alterations at the atrioventricular junction may also affect the cardiac conduction system. Displacement of the atrioventricular node or disruption of conduction pathway insulation can occur, producing conduction abnormalities that range from altered junctional rhythms to complete atrioventricular block [5].

Morphological Spectrum and Clinical Implications

The morphological spectrum includes partial, transitional and complete atrioventricular canal forms, each defined by specific patterns of annular orientation, leaflet bridging, chordal distribution and papillary muscle arrangement [13]. Recognition of these structural variations is important for evaluating volume overload, inflow obstruction and conduction vulnerability across the spectrum of atrioventricular canal and junctional malformations [30]. Table 3 demonstrates the major structural and functional implications of atrioventricular canal and junctional defects.

**Table 3:** Morphological elements in atrioventricular canal and junctional abnormalities Compiled by the authors based on information synthesized from [18,29,30]. The table is not directly reproduced from any single external source; therefore, permission from original publishers was not required. AVCD: atrioventricular canal defect, AV: atrioventricular, ASD: atrial septal defect, VSD: ventricular septal defect.

Canal Type	Primary Structural Feature	Valve Configuration	Hemodynamic Effect	Imaging Indicators	References
Partial AVCD	Incomplete cushion fusion	Separate AV valves with a cleft	Left-to-right shunting; regurgitation	Cleft leaflet; primum ASD	[29]
Transitional AVCD	Partial bridging leaflet	Shared subvalvular support	Mixed inflow imbalance	Asymmetric bridging pattern	[30]
Complete AVCD	Common AV junction	Single annulus with bridging leaflets	Large shunt; significant regurgitation	Common valve ring with VSD	[18]

Abnormalities of cardiac rotation, situs and laterality

Development of Cardiac Laterality and Situs

The establishment of cardiac rotation, situs orientation and laterality represents an early stage of embryonic patterning that determines the spatial organization of the atria, ventricles and great vessels [31]. These processes are regulated by left-right signaling pathways, ciliary motion at the embryonic node and morphogen gradients that guide asymmetric organ positioning [29]. Disturbances in these mechanisms alter the normal spatial relationships between cardiac chambers and thoracoabdominal organs, producing complex structural configurations [32].

Situs Variants and Atrial Isomerism

Situs inversus is characterized by mirror-image positioning of the heart and visceral organs, resulting in reversed chamber alignment and altered spatial relationships that may influence diagnostic interpretation and procedural access [17]. Atrial isomerism arises from incomplete lateralization and leads to duplication of either right-sided or left-sided atrial morphological features [29]. Right atrial isomerism typically presents with bilateral right atrial appendages, abnormal pulmonary venous connections, and complex outflow tract defects, often producing significant circulatory instability. Left atrial isomerism is associated with bilateral left atrial morphology, interrupted inferior vena cava patterns and atrioventricular conduction abnormalities [28].

Heterotaxy and Structural Consequences

Heterotaxy syndrome represents a broader spectrum of laterality disturbances involving abnormal arrangement of thoracic and abdominal organs, displaced venous pathways and altered cardiac looping patterns. These variations affect chamber alignment, systemic and pulmonary venous drainage and ventriculoarterial connections [14]. The position of the cardiac apex, the degree of atrial isomerism and the spatial orientation of the great arteries relative to the ventricles influence overall circulatory flow patterns [19].

Associated Structural Defects and Clinical Impact

Laterality disturbances frequently coexist with septation defects, outflow tract malalignment or atrioventricular valve abnormalities, further complicating cardiovascular hemodynamics [33]. Recognition of these structural relationships is essential for accurate morphological assessment and clinical management of laterality-related congenital heart disease [12].

Vascular ring and aortic arch variants as rare congenital entities

Developmental Basis of Aortic Arch Variants

Variants of the vascular ring and aortic arch arise from developmental alterations in the pharyngeal arch arteries, producing vascular structures that may encircle or compress the trachea and esophagus [34]. These anomalies occur when specific embryonic arches fail to regress or persist abnormally, creating atypical patterns of vascular encirclement [35].

Double Aortic Arch

In double aortic arch, both fourth pharyngeal arch derivatives persist, forming a complete vascular ring with variable dominance of the right or left arch limb [28]. This configuration can compress the trachea and esophagus circumferentially, producing symptoms related to airway obstruction or impaired swallowing as the child grows [9].

Aberrant Subclavian Artery Variants

The aberrant left subclavian artery is typically associated with a right aortic arch, where the vessel arises from a diverticulum or retroesophageal segment, often originating from a Kommerell diverticulum [32]. This configuration may produce anterior esophageal compression and may be associated with a ligamentous remnant completing the vascular ring [7]. Similarly, a left aortic arch with an aberrant right subclavian artery follows a retroesophageal course that alters the spatial relationship within the superior mediastinum and may produce feeding or respiratory symptoms in some individuals [18].

Additional Aortic Arch Variants

Other rare variants such as cervical aortic arch, intersecting arch patterns or abnormal ductal attachments further modify the arrangement of the great vessels and influence hemodynamic distribution within the thoracic inlet [30]. The severity of symptoms depends on the position, rigidity and angulation of the vascular segments relative to surrounding mediastinal structures [30].

Clinical and Morphological Significance

Accurate identification of aortic arch variants is important for understanding airway compression and circulatory alterations associated with vascular rings. Precise anatomical characterization also supports appropriate clinical evaluation and long-term management in individuals with aortic arch and vascular ring anomalies [36].

Integrative role of advanced imaging in defining cardiac morphology

Role of Advanced Imaging in Structural Assessment

Advanced imaging modalities play a critical role in evaluating the structural complexity of rare congenital heart diseases by providing high-resolution visualization of cardiac chambers, vascular connections and valvular anatomy [37]. Three-dimensional echocardiography enables dynamic assessment of cardiac structures, allowing accurate evaluation of valve leaflet motion, septal alignment and the spatial orientation of subvalvular components [38]. This technique also supports real-time assessment of morphological changes that influence inflow and outflow dynamics [24].

Prenatal and Cross-Sectional Imaging Techniques

Fetal echocardiography extends structural assessment into the prenatal period by detecting early abnormalities of cardiac looping, chamber formation and arterial alignment during gestation [39]. Computed tomography angiography provides detailed visualization of extracardiac pathways, coronary origins, aortic arch anatomy and relationships between great vessels and airway structures [20]. Its ability to generate three-dimensional reconstructions improves identification of anomalous vascular courses, complex venous connections and conotruncal malformations [8]. Cardiac magnetic resonance imaging adds detailed tissue characterization and volumetric analysis, enabling precise assessment of ventricular mass distribution, chamber interaction and intracardiac flow patterns [13].

Functional Interpretation and Hemodynamic Assessment

These imaging capabilities help clarify the functional consequences of structural abnormalities by demonstrating the degree of obstruction, regurgitation or intracardiac shunting associated with abnormal pathways [29].

Multimodality Imaging and Clinical Integration

Combining multiple imaging modalities enhances the overall interpretation of rare congenital cardiac anomalies [19]. Integrated evaluation allows detection of subtle structural imbalances, concealed vascular variants and complex morphological relationships that may not be visible with a single technique [26]. Comprehensive imaging also improves procedural planning by identifying surgical landmarks, ventriculo-arterial relationships and potential anatomical constraints imposed by surrounding structures [40].

Longitudinal Imaging and Clinical Monitoring

Long-term imaging follow-up provides important information on cardiac remodeling, postoperative adaptation and changes in chamber function over time [21]. High-resolution anatomical imaging therefore supports accurate diagnosis, informed clinical decision-making and improved understanding of structural determinants across the spectrum of rare congenital cardiac disorders [41].

Limitations and future recommendations

The current synthesis is limited due to heterogeneity of the offered morphological descriptions, variability among imaging modalities and scarce homogeneity in the categories of rare congenital cardiac disorders. Lack of consistency in the terminology of primary sources hampers direct comparison of structural patterns, and lack of complete developmental correlations hampers full interpretation of embryological pathways. Some of the conditions are still reported only by isolated clinical cases, which diminishes the richness of structural-functional correlations and limits the extrapolation of obtained results.

The recommendations for the future are to extend the standardized morphological formats that combine embryological sequences with the use of enhanced imaging standards, so as to interpret rare cardiac variants more consistently. The expansion of the multicenter databases that are harmonized in their diagnostic descriptors would enhance structural mapping and promote comparative evaluation across various populations. Improved applications of three-dimensional imaging, computational modelling and quantitative morphometry can sharpen the insight into subtle deviations of the anatomy. Further evolution of combined diagnostic pathways connecting morphology and clinical outcome would be useful in enhancing intervention planning, long-term follow-up and early identification of complicated congenital heart diseases.

## Conclusions

The morphology of rare congenital cardiac anomalies reflects intricate developmental processes that shape chamber formation, vascular arrangement and valvular patterning. Disturbances during these stages produce structural variations that alter circulatory dynamics, modify ventricular workload and influence long-term physiological stability. Understanding these developmental determinants enhances diagnostic interpretation, as even minor architectural differences can significantly affect disease severity and clinical presentation. Advances in imaging have greatly improved visualization of both intracardiac and extracardiac anatomy, enabling more accurate assessment of junctional alignment, vascular orientation and valvular function. When combined with embryological knowledge, imaging-based characterization provides valuable insight into expected hemodynamic behavior across different presentations. Recognizing these anatomical distinctions also supports more precise intervention planning by addressing both the primary defect and associated secondary changes. Consolidating morphological understanding across rare cardiac conditions contributes to a clearer conceptual framework of congenital heart structure and guides more effective management strategies. This integrated perspective ultimately promotes improved long-term outcomes for individuals with uncommon structural cardiac abnormalities.
